# Interactive effects of CO_2_ , temperature, and nitrate limitation on the growth and physiology of strain CCMP 1334 of the marine cyanobacterium *Synechococcus* (Cyanophyceae)

**DOI:** 10.1111/jpy.13531

**Published:** 2024-11-30

**Authors:** Alyssa K. Sharbaugh, Edward A. Laws

**Affiliations:** ^1^ Department of Environmental Sciences Louisiana State University Baton Rouge Louisiana USA

**Keywords:** acclimation, algal model, climate change

## Abstract

The marine cyanobacterium *Synecococcus* sp. (CCMP 1334) was grown in a continuous culture system on a 12:12 h light:dark cycle at all combinations of low and high pCO_2_ (400 and 1000 ppmv, respectively), nutrient availability (nitrate‐limited and nutrient‐replete conditions), and temperatures of 21, 24, 28, 32, and 35°C. The maximum nutrient‐replete growth rate was ~1.15 day^−1^ at 32–35°C. Median nutrient‐replete growth rates were higher at 1000 ppmv than at 400 ppmv pCO_2_ at all temperatures. Carbon:nitrogen ratios were independent of pCO_2_ at a fixed relative growth rate (i.e., growth rate ÷ nutrient‐replete growth rate) but decreased with increasing temperature. Carbon:chlorophyll *a* ratios were decreased monotonically with increasing temperature and were higher under nitrate‐limited than nutrient‐replete conditions. Ratios of phycoerythrin to chlorophyll *a* were independent of growth conditions. Productivity indices were independent of temperature and nutrient limitation but were consistently higher at 1000 ppmv than 400 ppmv pCO_2_. Both growth rates and dark respiration rates were positively correlated with temperature, and the associated *Q*_10_ values were 2.2 and 2.3, respectively. A model of phytoplankton growth in which cellular carbon is allocated to structure, storage, or the light or dark reactions of photosynthesis accounted for the general patterns of cell composition and growth rate. This strain of *Synechococcus* appears well suited to changes in environmental conditions that are expected as the climate warms in response to anthropogenic emissions of CO_2_.

**Abbreviations**
Chl a: chlorophyll a
DIC: dissolved inorganic carbon
PC: particulate carbon
PE: phycoerythrin
PI: productivity index
PN: particulate nitrogen
PQ: photosynthetic quotient
TA: total alkalinity

## INTRODUCTION

Phytoplankton are responsible for about half of global primary production (Field et al., 1998). *Synechococcus* is one of the most abundant genera of marine phytoplankton and can be observed anywhere from the equator to polar waters. It is the dominant picophytoplankton in coastal waters and temperate/mesotrophic waters of the open ocean (Partensky et al., 1999). Its principal photosynthetic pigment is chlorophyll *a*, and its main light‐harvesting antennae, phycobilisomes, are nitrogen‐rich protein‐pigment complexes that allow the cells to capture energy at wavelengths outside the principal absorption bands of chlorophyll *a* (Mackey et al., 2013).

Carbon dioxide is a key component of the global carbon cycle, the balance of which has been disrupted by anthropogenic combustion of fossil fuels. Scientists predict that the partial pressure of CO_2_ in the atmospheric (pCO_2_) may reach 1000 ppmv by the end of this century (Cheng et al., 2022). The exchange of CO_2_ between the atmosphere and ocean surface will then result in a decline of the pH of the surface ocean by 0.3 (Yool et al., 2013). Because CO_2_ absorbs infrared radiation emitted by Earth, the increase in atmospheric pCO_2_ is expected to increase sea surface temperatures by 1–4°C (Feng et al., 2009). The associated increase in thermal stratification is expected to lead to a shoaling of the mixed layer and increase in the gradient of the nutricline in the subtropical gyres that will reduce the influx of nutrients through the nutricline and increase the degree of nutrient limitation of biomass in the euphotic zone (Beardall et al., 2009; Rost et al., 2008). Climate change effects are thus expected to cause simultaneous changes in the physicochemical environment of the mixed layer in terms of temperature, pCO_2_, pH, and the supply of inorganic nutrients.

Several studies on the effects of environmental conditions on the growth and physiology of *Synechococcus* sp. CCMP 1334 have addressed the effects of temperature and CO_2_ partial pressure (Fu et al., 2007) as well as nitrate limitation (Liu et al., 1999; Ruan et al., 2018). However, no study has examined the direct and interactive effects of simultaneous changes of temperature, pCO_2_, and nitrate‐limitation.

This study was undertaken to determine the direct and interactive effects of such simultaneous changes in environmental conditions on a species of the genus *Synechococcus* originally isolated in 1980 from the Sargasso Sea and maintained in the Center for the Culture of Marine Phytoplankton (CCMP) as strain CCMP 1334. The genus *Synechococcus* has been divided into more than 20 genetically distinct clades (Harcourt et al., 2024; Lee et al., 2019; Sohm et al., 2016), of which strain CCMP 1334 (WH 7803) belongs to clade V. This strain is considered to be representative in many respects of the *Synechococcus* clades observed in mesotrophic areas of the ocean, but it differs from most other species of *Synechococcus* in that is possesses two glutamine synthetases (Domínguez‐Martín et al., 2016).

Previous studies of CCMP 1334 (DC2, WH 8102) have included Morris and Glover (1981), Barlow and Alberte (1985), Kana and Glibert (1987a, 1987b), Fu et al. (2007), Li et al. (2019), Bao and Gao (2021), and Basu and Mackey (2022). Morris and Glover (1981) observed the maximum growth rate at 20°C and continuous illumination with cool‐white, fluorescent light to be 1.2 day^−1^ and that the growth rate became light‐saturated at an irradiance of ~40 μmol photons · m^−2^ · s^−1^. Barlow and Alberte (1985) observed that cultures acclimated to irradiances of no more than 25 μmol photons · m^−2^ · s^−1^ were light‐inhibited when exposed to irradiances greater than 180 μmol photons · m^−2^ · s^−1^, but there was no evidence of light inhibition by irradiances as high as 1080 μmol photons · m^−2^ · s^−1^ if the cells were acclimated to irradiances of 100 or 250 μmol photons · m^−2^ · s^−1^. Kana and Glibert (1987a, 1987b) acclimated cells to continuous irradiance of 30–2000 μmol photons · m^−2^ · s^−1^ from cool‐white fluorescent lights at a temperature of 22 to 23°C. They allowed at least 2 weeks for the cells to acclimate to each irradiance. They reported maximum growth rates of 1.7–1.9 day^−1^ at irradiances greater than ~300 μmol photons · m^−2^ · s^−1^ and saw no evidence of light inhibition at irradiances as high as 2000 μmol photons · m^−2^ · s^−1^. Fu et al. (2007) grew their cells on a 12:12 light:dark (L:D) cycle and at an irradiance of 45 μmol photons · m^−2^ · s^−1^ at temperatures of either 20 or 24°C and CO_2_ partial pressures of either 380 or 750 ppmv. They observed that growth rates responded positively to an increase in either temperature or pCO_2_ but that the effect of increasing the temperature was greater than the effect of increasing the pCO_2_. They reported a growth rate of 0.72 day^−1^ at 24°C and CO_2_ partial pressure of 750 ppmv. Li et al. (2019) grew their cultures at temperatures of 22–28°C and an irradiance of 20 μmol photons · m^−2^ · s^−1^ on a 14:10 L:D cycle of illumination. They observed that the optimum temperature for growth was 25°C and reported maximum growth rates of 0.62–0.67 day^−1^ at that temperature. Bao and Gao (2021) grew their cells at 23.5°C in 100‐mL quartz tubes at an irradiance of 6 μmol photons · m^−2^ · s^−1^ provided on a 12:12 L:D cycle of illumination with CO_2_ partial pressures of either 400 or 1000 ppmv. They reported a maximum growth rate of ~0.54 day^−1^ at an irradiance of 25 μmol photons · m^−2^ · s^−1^ and observed that growth rates were inhibited by higher irradiances. Increasing the CO_2_ partial pressure from 400 to 1000 ppmv increased growth rates by no more than ~2.5%. Basu and Mackey (2022) grew their cultures at temperatures of 22, 24, and 26°C and CO_2_ partial pressures of 400, 600, and 800 ppmv. The cells were illuminated continuously at an irradiance of 20 μmol photons · m^−2^ · s^−1^. They reported growth rates that ranged from 0.25 to 0.40 day^−1^. The growth rates were highest at 22°C and 600 ppmv CO_2_ and lowest at 26°C and 800 ppmv CO_2_.

The implication of these previous studies is, not surprisingly, that how CCMP 1334 responds to an increase of temperature or pCO_2_ depends on the conditions to which it is acclimated and the time allowed for acclimation. Given sufficient time for acclimation, CCMP 1334 appears to respond positively to an increase of irradiance over the range of irradiances likely to be encountered in the ocean (Kana & Glibert, 1987a, 1987b). The effect of temperature is unclear because one study reported a positive correlation between growth rates and temperatures of no more than 24°C (Fu et al., 2007), whereas other studies have reported that growth rates reach maximums at a temperature of 22°C (Basu & Mackey, 2022) or 25°C (Li et al., 2019).

One goal of this study was, therefore, to carefully examine the effects of temperature on CCMP 1334, including both growth rates and respiration rates. Because none of the previous studies has considered the effects of nutrient limitation and because climate change is expected to lead to a reduction of the influx of nutrients through the nutricline due to shoaling of the mixed layer (Beardall et al., 2009; Rost et al., 2008), we grew CCMP 1334 under both nutrient‐replete and nitrate‐limited conditions in a continuous culture system.

To address these general issues, we set up experiments to test several specific null hypotheses:

- The nutrient‐replete growth rates of *Synechococcus* sp. CCMP 1334 are unaffected by changing the pCO_2_ from 400 to 1000 ppmv
- If pCO_2_ affects growth rates, then the effect is independent of temperature.
- The carbon:nitrogen (C:N) ratio of cells is independent of both temperature and pCO_2_ at a fixed relative growth rate (i.e., the ratio of the nutrient‐limited growth rate relative to the nutrient‐replete growth rate under otherwise identical conditions). According to Goldman (1980), the C:N ratio is a unique function of the relative growth rate.
- Variations of the C:N ratio, the chlorophyll *a*:carbon (chl *a*:C) ratio, and the phycoerythrin:carbon (PE:C) ratio are independent of pCO_2_ and can be explained as functions of temperature and nutrient limitation according to a model of carbon allocation by Shuter (1979) and Laws & Chalup (1990).
- The productivity index will be lower under nitrate‐limited than nutrient‐replete conditions but will be unaffected by either temperature or pCO_2_.

## MATERIALS AND METHODS

### Culturing conditions

**FIGURE 1:**
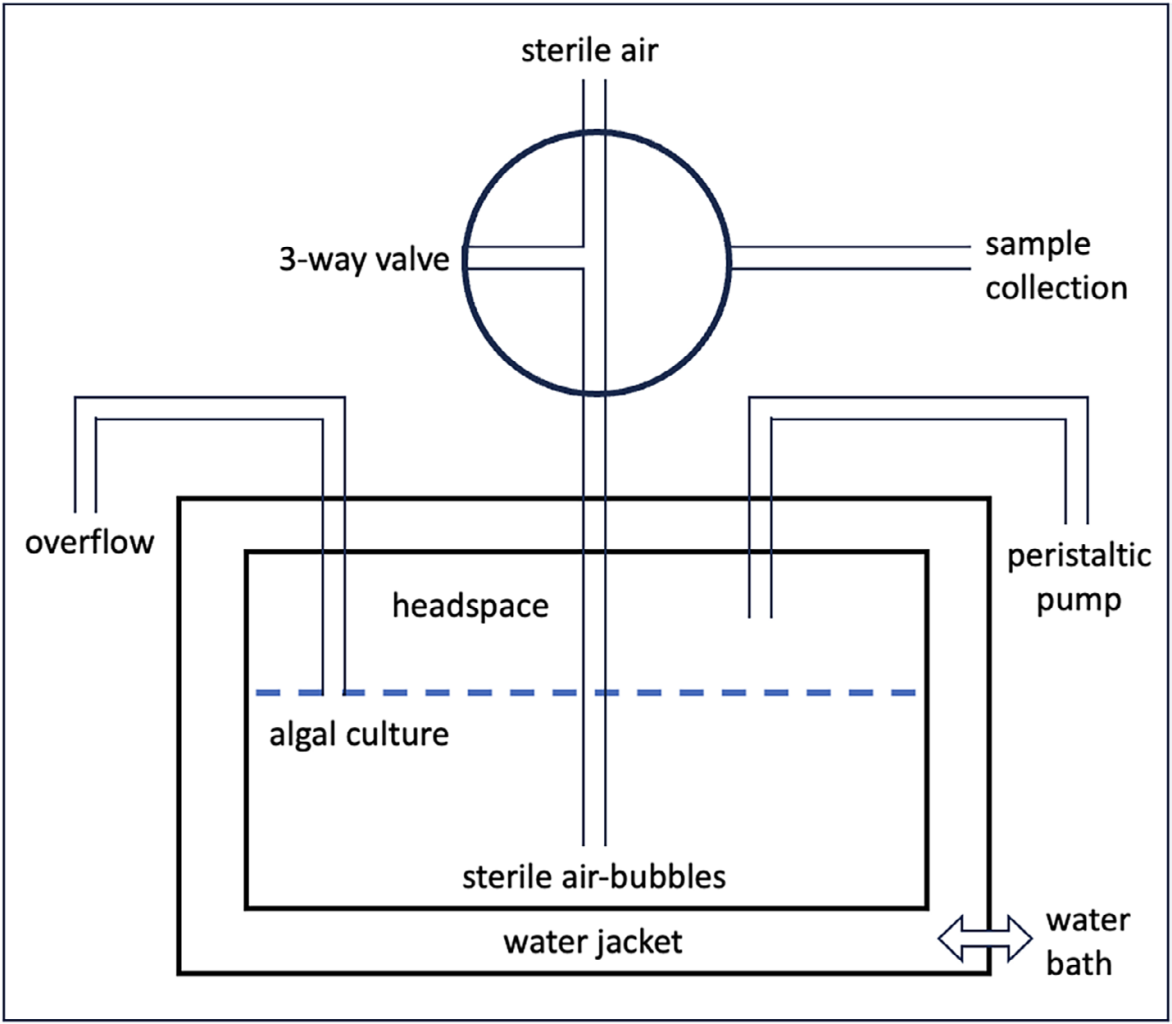
Diagram of continuous culture system showing configuration of peristaltic pump, overflow, and three‐way valve used to bubble air and collect samples.

The culture of *Synechococcus* sp. CCMP 1334 was obtained from the National Center for Marine Algae and Microbiota (Bigelow Laboratories) and had been originally isolated from the Sargasso Sea. It was grown in a continuous culture system (Figure 1) identical to that described by Laws and McClellan (2022). The experiments were conducted from October 2021 through December 2023 and included a total of 20 steady states at five temperatures (21, 24, 28, 32, and 35°C) and low and high pCO_2_ (400 and 1000 ppmv, respectively) and either nitrate‐limited or nutrient‐replete growth conditions (44 and 880 μM NaNO_3_, respectively). Light was provided by a bank of light‐emitting diode lamps at an irradiance of 150 μmol photons · m^−2^ · s^−1^ of visible light (400–700 nm wavelength) measured with a quantum scalar light meter (Biospherical Instruments Model QSL 2100, San Diego, CA, USA) during the photoperiod. The light and dark periods were 12 h each.

The double walled design of the growth chamber allowed water from a temperature‐regulated (±0.1°C) water bath to be circulated between the inner and outer walls for temperature control. Sterile‐filtered air (0.2‐μm filter) was continuously bubbled into the chemostat to drive the overflow and maintain the partial pressure of CO_2_. The air had a pCO_2_ of either 400 or 1000 ppmv to simulate ambient or elevated CO_2_ partial pressures, respectively. For nutrient‐replete conditions, the medium was pumped into the growth chamber via a peristaltic pump at approximately the same rate as the population growth rate to maintain a relatively constant (±5%) optical density (OD). After establishing an average growth rate under nutrient‐replete conditions, nitrate‐limited medium was then pumped into the growth chamber at approximately half the nutrient‐replete rate to create nitrate‐limited conditions. The culture was stirred by a Teflon‐coated magnetic stir bar. The medium consisted of inorganic seawater salts sufficient to produce a salinity of 35 and a total alkalinity of 2.4 meq · kg^−1^. Vitamins and silicate were not added. The medium consisted of deionized water, Instant Ocean (27.6 g · L^−1^), artificial seawater salts to produce a total alkalinity of 2.4 meq · kg^−1^ (NaCl: 5.05 g · L^−1^, Na_2_SO_4_: 0.846 g · L^−1^, KCl: 0.148 g · L^−1^, KBr: 0.0211 g · L^−1^, H_3_BO_3_: 0.00634 g · L^−1^, MgCl_2_ **·**6H_2_O: 2.28 g · L^−1^, CaCl_2_ **·**2H_2_O: 0.317 g · L^−1^, SrCl_2_ **·**6H_2_O: 0.00423 g · L^−1^) nitrate (880 μM NaNO_3_ for nutrient‐replete and 44 μM for nitrate‐limited), phosphate (36.6 μM NaH_2_PO_4_ **·**H_2_O), and trace metals, as per Sunda and Hardison (2007).

### Inorganic carbon system

For each set of conditions (pCO_2_, nitrate concentration, and temperature), the pCO_2_ of the chemostat was monitored to ensure alignment with experimental goals. The pCO_2_ was calculated at the beginning and end of the photoperiod because photosynthesis took up CO_2_ during the photoperiod, and CO_2_ was respired during the scotoperiod. Cell density was limited by the pumping rate to ensure that biological activity did not greatly alter the pCO_2_ in the growth chamber.

The pH was measured on the total hydrogen ion scale using the spectrophotometric method described by Dickson et al. (2009) in SOP6b. The absorbances for pH determinations were measured on a Spectronic Helios Delta UV–Visible spectrophotometer (Thermo Scientific, Waltham, MA, USA). The total alkalinity (TA) was calculated using a single‐point titration method described by Breland and Byrne (1993). After the pH and TA had been determined, we used the salinity, temperature, and equations in Zeebe and Wolf‐Gladrow (2001) to calculate the concentrations of carbonic acid (H_2_CO_3_), dissolved inorganic carbon (DIC), and pCO_2_ in the growth chamber.

### Sampling and analysis

The growth rate of *Synechococcus* was calculated at approximately the same time each day. This calculation required knowledge of the dilution rate and change in OD from one day to the next. The dilution rate was measured by determining the volume of medium in the overflow flask relative to the volume of the growth chamber.

A Spectronic Helios model Delta spectrophotometer (Thermo Fisher) was used to measure the OD each day. After zeroing with deionized water, light at a wavelength of 750 nm was passed through a cuvette containing growth chamber medium. We assumed that the OD measured in this way was proportional to the concentration of cells because there is no light absorption at 750 nm (Maerker & Szekielda, 1976), and the attenuation of light at 750 nm is due entirely to scattering. Given the daily dilution rate (D) and the initial OD and OD 1 day later (final), Equation 1 was used to calculate daily growth rate.

(1) $$\text{Growth rate}\;\left({\mathrm{day}}^{-1}\right)=\ln\left({\mathrm{OD}}_{\text{final}}/{\mathrm{OD}}_{\text{initial}}\right)+D$$

Sampling for variables other than growth rate did not begin until the chemostat was in a steady state (i.e., four doubling times had passed since the last change of conditions). The sampling consisted of filtering growth chamber medium through glass fiber filters (GFFs) or polycarbonate membrane filters. Samples were collected at the beginning and end of seven photoperiods for each set of conditions. The GFFs were used for chlorophyll and elemental analysis, and polycarbonate membranes were used to measure phycoerythrin.

To measure chlorophyll concentrations, GFF filters were placed in methanol (MeOH) overnight at −7°C. The filter debris was separated from the MeOH by filtration through a GFF filter, and the absorption of chlorophyll *a* in the clarified MeOH was read in a spectrophotometer at a wavelength of 664 nm with a correction for any scattering at 750 nm. The following equation from Holm‐Hansen and Riemann (1978) was used to calculate the concentration of chlorophyll *a*.

(2) $$\mathrm{chl}\;a\;\left(g\cdot L^{-1}\right)=\frac{A_{664}-A_{750}}{74.75\;L\times g^{-1}\times {\mathrm{cm}}^{-1}}\times \frac{V_{\text{MeOH}}}{V_{f}}$$

where L is the path length of the cuvette (cm), *V*_MeOH_ is the volume of methanol used (mL), and *V*_f_ is the volume of sample filtered (mL).

Concentrations of particulate carbon (PC) and particulate nitrogen (PN) were measured using an Exeter Elemental Analyzer (model CE 440; Exeter Analytical, Chelmsford, MA, USA). After calibrating the instrument with blanks and acetanilide standards, collected filters were combusted in the elemental analyzer to determine carbon and nitrogen contents.

The following equation from Laws et al. (2020) was used to calculate the dark respiration rate (R) in units of per day.

(3) $$R\;\left({\mathrm{day}}^{-1}\right)=2\;\ln\left(C_{12}/C_{0}\right)-D$$

where C_12_ and C_0_ are the concentrations of particulate carbon at the end and beginning of the photoperiod, respectively.

The photosynthetic rate (P) during the photoperiod was calculated using Equation 4 taken from Laws et al. (2020). The daily dilution rate (D) in the exponents in Equation 4 is multiplied by 0.5, which is the duration of the photoperiod in days.

(4) $$P\;\left(g\;C\cdot h^{-1}\right)=\left(\frac{C_{12}-C_{0}\times e^{-0.5\times D}}{1-e^{-0.5\times D}}\right)\times D/24$$

The productivity index (PI), or photosynthetic rate normalized to chlorophyll *a*, was then calculated by dividing the photosynthetic rate (Equation 4) by the average of the chlorophyll concentrations calculated at the beginning and end of the photoperiod. The units for productivity index were therefore g C · g^−1^ chl *a* · h^−1^.

To measure concentrations of phycoerythrin, polycarbonate filters were digested in a buffer consisting of 10 mM Na_3_PO_4_, 150 mM NaCl, and lysozyme (10 mg lysozyme per 1 mL of buffer). The samples were then incubated at 37°C overnight to allow for cell lysis. After passing the buffer through a polycarbonate membrane, the absorption of phycoerythrin was measured with the spectrophotometer at a wavelength of 543 nm with a correction for any scattering at 750 nm. The phycoerythrin concentration was calculated using the following equation from Wyman (1992).

(5) $$\mathrm{PE}\;\left(g\cdot L^{-1}\right)=\frac{A_{543}-A_{750}}{9.43L\times g^{-1}\times {\mathrm{cm}}^{-1}}\times \frac{V_{b}}{V_{f}}$$

where *L* is the path length of the cuvette (cm), *V*_b_ is the volume of buffer used (mL), and *V*_f_ is the volume of sample filtered (mL).

### Statistics

We used parametric statistical tests (e.g., paired *t*‐tests) to detect treatment effects if the data satisfied the assumptions of normality and homoscedasticity. We used a Shapiro–Wilk test for normality and Bartlett's test for homoscedasticity. We accepted the null hypothesis if the type I error rates exceeded 0.05. If the data failed either of the tests for normality or homoscedasticity, we used non‐parametric statistical tests (e.g., Kruskal–Wallis test).

## RESULTS

Table 1 provides a summary of the results obtained at all 20 steady states. Although the growth chamber was continuously bubbled with air containing either 400 or 1000 ppmv pCO_2_, the pCO_2_ in the growth chamber varied throughout the day due to biological activity. The phytoplankton took up CO_2_ during the photoperiod and respired CO_2_ during the scotoperiod. When the pCO_2_ in the air was 400 ppm, the pCO_2_ in the growth chamber varied between 335 ± 86 ppmv (end of photoperiod) and 468 ± 29 ppmv (beginning of photoperiod). When the pCO_2_ in the air was 1000 ppm, the pCO_2_ in the growth chamber varied between 879 ± 81 ppmv (end of photoperiod) and 1107 ± 119 ppmv (beginning of photoperiod). The values in Table 1 are the medians (±median absolute deviations) of all values calculated based on pH and total alkalinity measurements.

**TABLE 1:** Summary of growth conditions and results of continuous culture experiments with *Synechococcus* strain CCMP 1334.

Nitrate availability	pCO_2_	Temp (°C)	Growth rate (day^−1^) median (mad)	TA (meq · kg^−1^)	pH	Carbon:Nitrogen (g C · g N), lights off, lights on	Carbon:Chl *a* (g C · g^−1^ chl *a*) lights off, lights on	Phycoerythrin:Chl *a* (g PE · g^−1^ chl *a*) lights off, lights on	Productivity index (g C · g^−1^ chl *a* · h^−1^) median (mad)	Respiration rate (day^−1^) median (mad)
Nutrient‐Replete	400 ppmv	21	0.336 (0.101)	2.43	8.07	6.85, 6.14	190, 171	5.93, 7.66	6.33 (1.79)	0.117 (0.0127)
		24	0.421 (0.141)	2.46	8.07	7.10, 6.41	204, 170	4.23, 4.95	7.64 (0.625)	0.125 (0.0239)
		28	0.606 (0.0878)	2.53	8.07	5.52, 4.52	127, 109	2.18, 1.20	6.26 (0.319)	0.264 (0.0574)
		32	1.11 (0.203)	2.49	8.05	5.38, 4.58	135, 120	1.00, 1.19	5.95 (1.46)	0.260 (0.0664)
		35	1.03 (0.132)	2.51	8.05	5.71, 4.93	93, 105	4.05, 3.13	5.74 (2.13)	0.250 (0.0905)
	1000 ppmv	21	0.390 (0.111)	2.44	7.73	6.80, 6.21	185, 153	5.84, 4.37	7.12 (1.14)	0.0946 (0.0100)
		24	0.476 (0.226)	2.44	7.73	6.78, 5.50	126, 109	5.47, 3.98	8.23 (3.10)	0.242 (0.0600)
		28	0.717 (0.0868)	2.44	7.73	6.46, 4.94	113, 91	9.51, 13.45	7.37 (1.55)	0.336 (0.0528)
		32	1.12 (0.328)	2.43	7.72	6.24, 4.65	87, 68	3.67, 3.85	9.59 (1.71)	0.349 (0.0624)
		35	1.17 (0.195)	2.45	7.72	5.47, 4.41	72, 65	4.35, 1.90	8.69 (1.85)	0.356 (0.109)
Nitrate‐limited	400 ppmv	21	0.193 (0.101)	2.42	8.07	8.53, 7.77	405, 254	3.85, 3.08	5.13 (1.24)	0.0828 (0.0226)
		24	0.176 (0.0259)	2.43	8.06	8.39, 7.39	324, 302	4.62, 4.67	6.07 (1.18)	0.114 (0.0276)
		28	0.243 (0.0359)	2.45	8.06	9.13, 7.49	204, 178	5.11, 6.36	5.16 (0.594)	0.196 (0.0331)
		32	0.662 (0.0783)	2.45	8.05	8.40, 5.75	149, 128	6.32, 7.48	8.19 (0.720)	0.131 (0.0274)
		35	0.514 (0.0752)	2.45	8.04	7.43, 5.37	126, 87	4.64, 4.85	5.85 (0.276)	0.236 (0.0271)
	1000 ppmv	21	0.208 (0.0761)	2.42	7.73	8.83, 8.24	550, 472	No data, No data	8.90 (2.11)	0.0706 (0.00750)
		24	0.195 (0.0426)	2.43	7.73	8.30, 7.28	287, 247	4.11, 4.36	6.78 (0.984)	0.128 (0.0224)
		28	0.251 (0.0482)	2.45	7.73	8.82, 7.21	207, 172	5.17, 4.72	5.33 (0.332)	0.167 (0.0105)
		32	0.611 (0.107)	2.45	7.72	6.97, 5.09	110, 101	5.15, 7.14	6.60 (0.347)	0.226 (0.0326)
		35	0.535 (0.0694)	2.45	7.72	7.66, 5.28	131, 113	4.75, 5.76	6.50 (0.713)	0.326 (0.0399)

Abbreviation: mad, median absolute deviation.

### Growth rates

**FIGURE 2:**
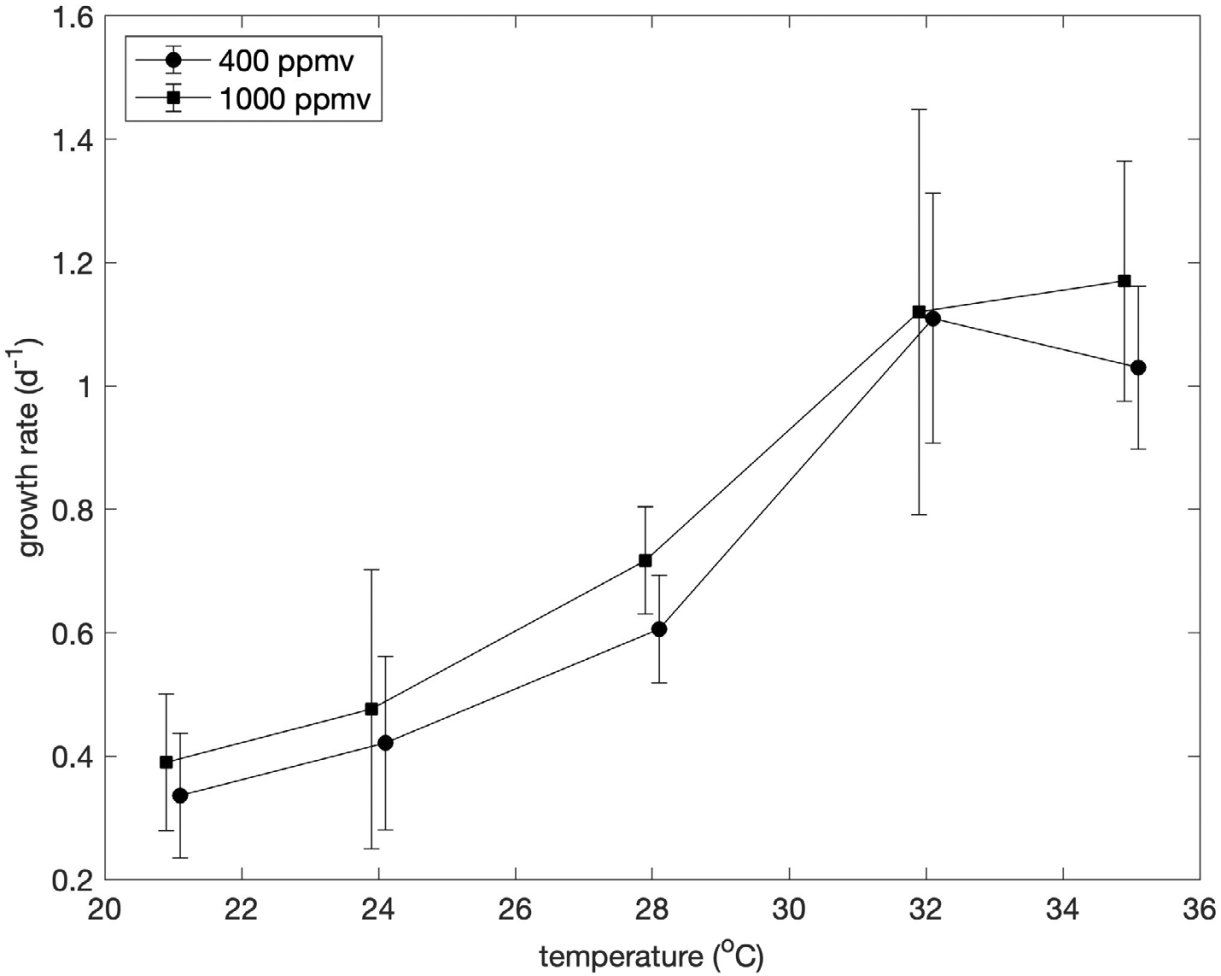
Nutrient‐replete growth rates (day^−1^) of *Synechococcus* as a function of temperature under conditions of 400 ppmv pCO_2_ and 1000 ppmv pCO_2_. Values are medians of 9–45 measurements made on consecutive days at the same time during the photoperiod. Error bars show the median absolute deviations. Data are offset by ±0.1°C from true temperatures to make error bars clear.

Under nutrient‐replete conditions (Figure 2), the median (±median absolute deviation) growth rates of *Synechococcus* ranged from 0.336 ± 0.101 day^−1^ at 21°C and ambient pCO_2_ to 1.17 ± 0.195 day^−1^ at 35°C and elevated pCO_2_. Temperatures of 21, 24, and 28°C were suboptimal, and growth rates peaked between 32 and 35°C. A Kruskal–Wallis one‐way analysis of variance (ANOVA) of the growth rates confirmed that there was a significant difference in nutrient‐replete growth rates at different temperature and pCO_2_ combinations (*p* = 7.70 × 10^−34^).

At all five temperatures tested, the median nutrient‐replete growth rate was higher at 1000 ppmv pCO_2_ than at 400 ppmv pCO_2_ (*p* = 0.0625, sign test). The differences were significant at 28 and 35°C based on a Kruskal–Wallis test at *p* < 0.03, and the difference was significant at 24°C based on Welch's *t*‐test at *p* = 0.02. We therefore rejected our first null hypothesis that changing the pCO_2_ from 400 to 1000 ppmv would have no effect on the growth rates of *Synechococcus* but with the caveat that the effect was small and not statistically significant at two of the five temperatures.

### Carbon:nitrogen ratios

**FIGURE 3:**
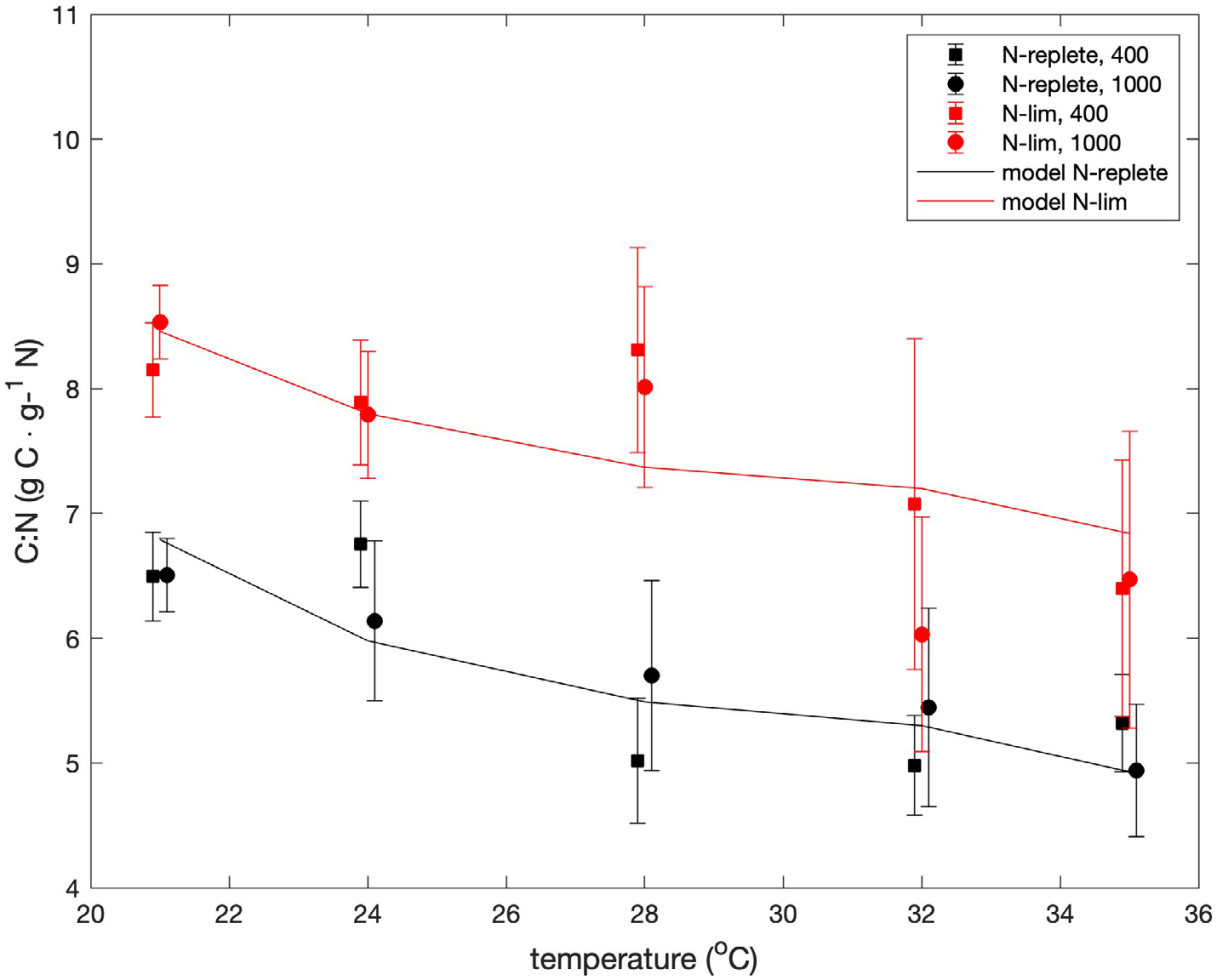
C/N ratios versus temperature. Symbols show averages of measurements made at the beginning and end of the 12‐h photoperiod. Squares and circles indicate results at pCO_2_ values of 400 and 1000 ppmv, respectively. Upper and lower bounds are the median ratios at the end and beginning of the photoperiod, respectively. Data are offset by ±0.1°C from true temperatures to make error bars clear.

Figure 3 shows the C:N ratios as functions of temperature, nutrient limitation, and pCO_2_. Based on the findings of Goldman (1980), we hypothesized that the C:N ratios would be constant at the same relative growth rate. The relative growth rate defined by Goldman (1980) is the nutrient‐limited growth rate divided by nutrient‐replete growth rate under otherwise identical conditions (i.e., temperature and pCO_2_). In our experiments, the relative growth rates were 1.0 and ~0.5 for the nutrient‐replete and nitrate‐limited studies, respectively. A Kruskal–Wallis (KW) test revealed no significant effect of pCO_2_ on the C:N ratios (*p* = 0.766), and as expected based on the twofold difference in relative growth rates, the KW test revealed a significant difference between C:N ratios under nutrient‐replete and nitrate‐limited conditions (*p* = 6 × 10^−5^). However, the Pearson's correlation coefficient between temperatures and C:N ratios was negative and significantly different from zero under both nutrient‐replete (*p* = 0.0011) and nitrate‐limited (*p* = 0.0048) conditions. We therefore rejected the hypothesis that the C:N ratios would be independent of temperature at a fixed relative growth rate.

### Metrics normalized to Chl *a*

**FIGURE 4:**
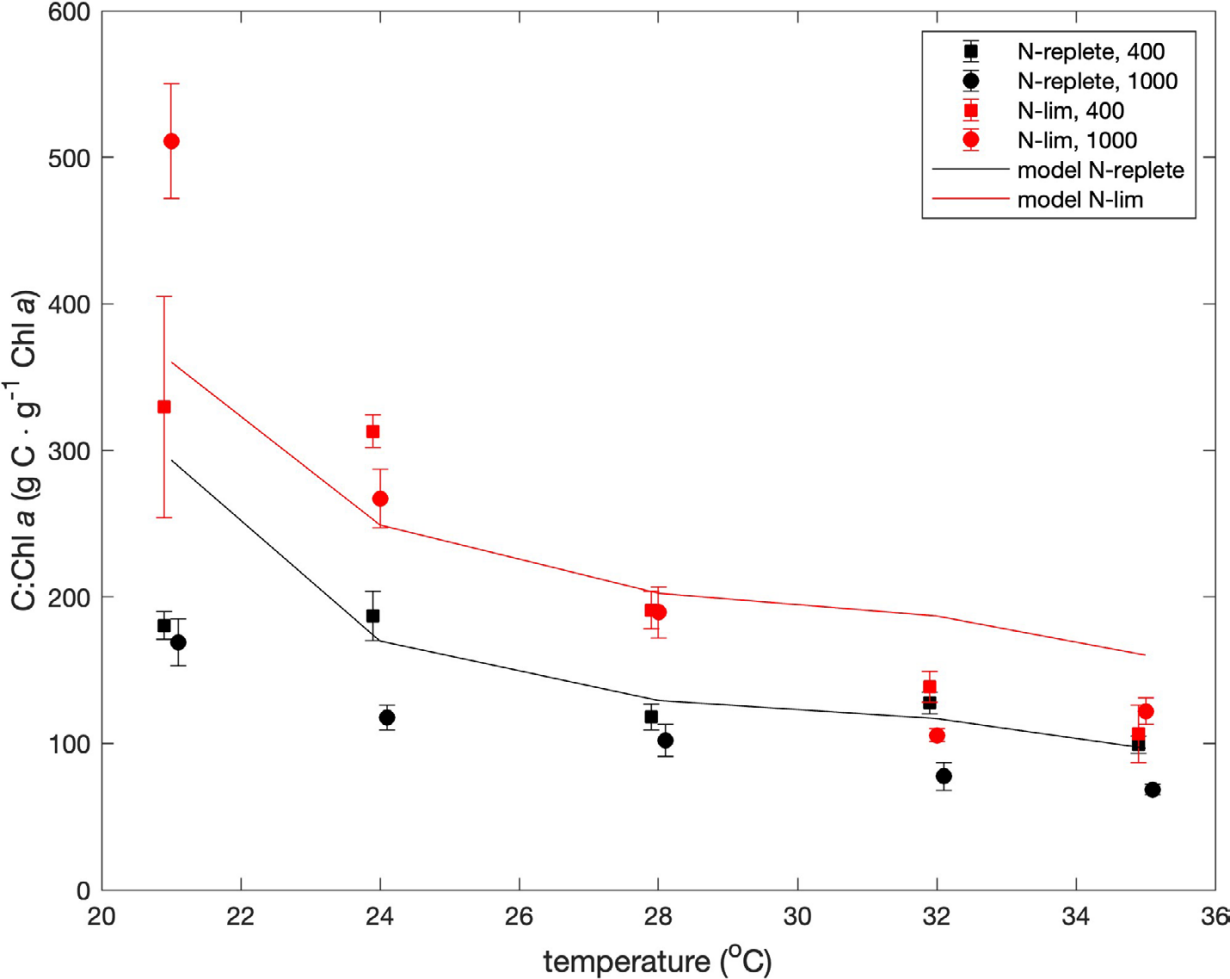
C:chl *a* ratios versus temperature. Symbols show averages of measurements made at the beginning and end of the 12‐h photoperiod. Squares and circles indicate results at pCO_2_ values of 400 and 1000 ppmv, respectively. Upper and lower bounds are the median ratios at the end and beginning of the photoperiod, respectively. Data are offset by ±0.1°C from true temperatures to make error bars clear.

Figure 4 shows the C:Chl *a* ratios as functions of temperature, nutrient limitation, and pCO_2_. A Kruskal–Wallis (KW) test revealed no significant effect of pCO_2_ on the C:Chl *a* ratios (*p* = 0.23) but a significant difference (*p* = 0.002) between the ratios under nutrient‐replete and nitrate‐limited conditions. The C:Chl *a* ratios were clearly higher under nitrate‐limited conditions at temperatures of 21–28°C, but at 32 and 35°C, the difference was smaller and less significant (KW test, *p* = 0.059). The Pearson correlation coefficient between temperatures and C:Chl *a* ratios was negative and significantly different from zero under both nutrient‐replete (*p* = 2.3 × 10^−5^) and nitrate‐limited (*p* = 3.0 × 10^−7^) conditions.

**FIGURE 5:**
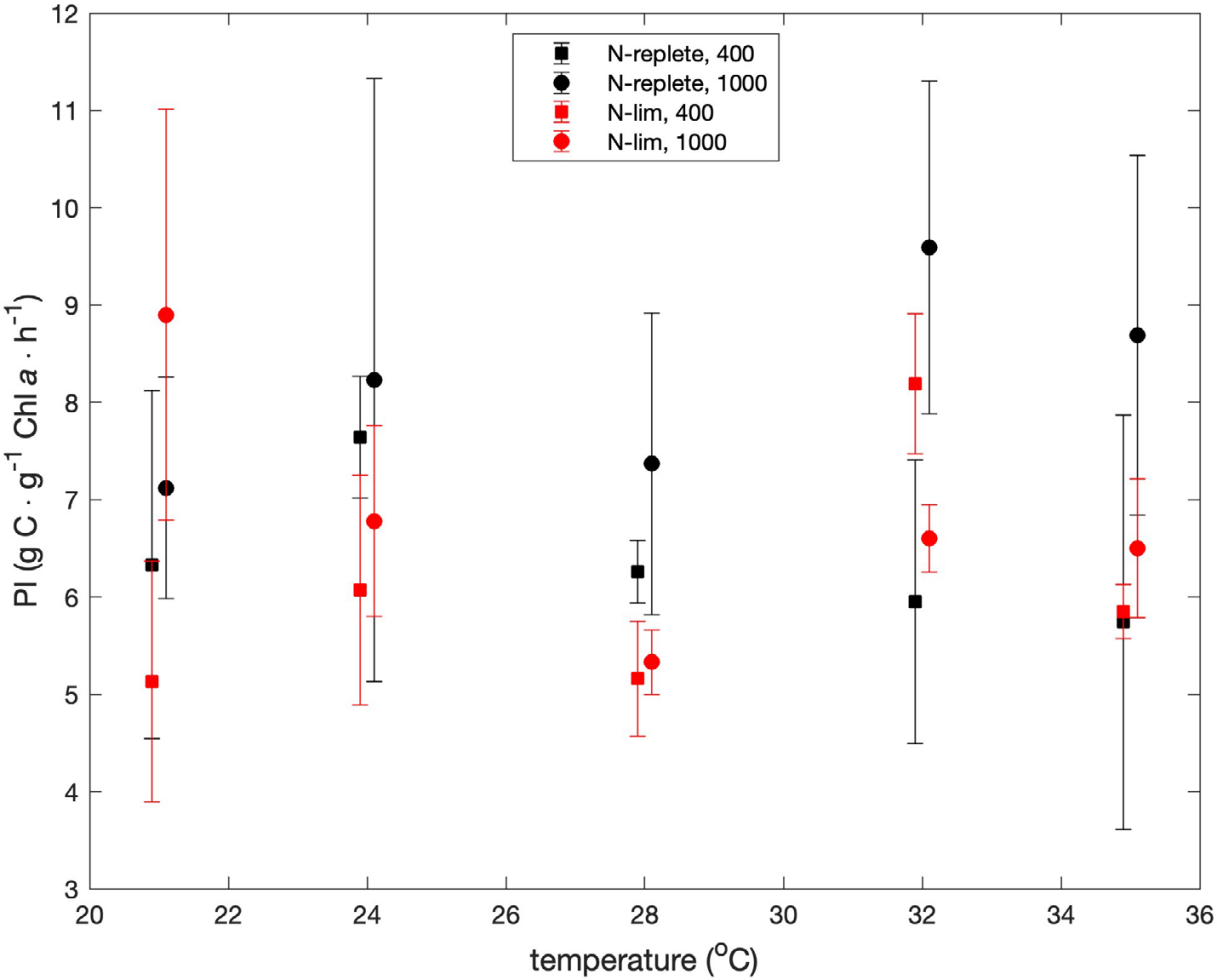
Productivity indices as functions of temperature and nutrient limitation. Symbols show averages of measurements made at CO_2_ partial pressures of 400 and 1000 ppmv. Error bars show the range of values at 400 and 1000 ppmv. Squares and circles indicate results at pCO_2_ values of 400 and 1000 ppmv, respectively. Data are offset by ±0.1°C from true temperatures to make error bars clear.

Figure 5 shows Productivity indices (PIs) as a function of temperature, pCO_2_, and degree of nutrient limitation. A KW test revealed a significant difference (*p* = 0.0156) in PIs measured at pCO_2_ values of 400 and 1000 ppmv. However, KW tests revealed no significant difference between nutrient‐replete and nitrate‐limited PIs (*p* = 0.15) and no significant effect of temperature (*p* = 0.52). The average PIs at pCO_2_ values of 400 and 1000 ppmv were 6.01 and 7.25 g C · g^−1^ chl *a* · h^−1^.

A KW test revealed no effect of temperature on PE:Chl *a* ratios (*p* = 0.5), and paired *t*‐tests revealed no effect of nutrient limitation (*p* = 0.82) or pCO_2_ (*p* = 0.11) on PE:Chl *a* ratios (see Table 1). The median ± median absolute deviation of the PE:Chl *a* ratios was 4.5 ± 0.48 g · g^−1^.

### Dark respiration rates

**FIGURE 6:**
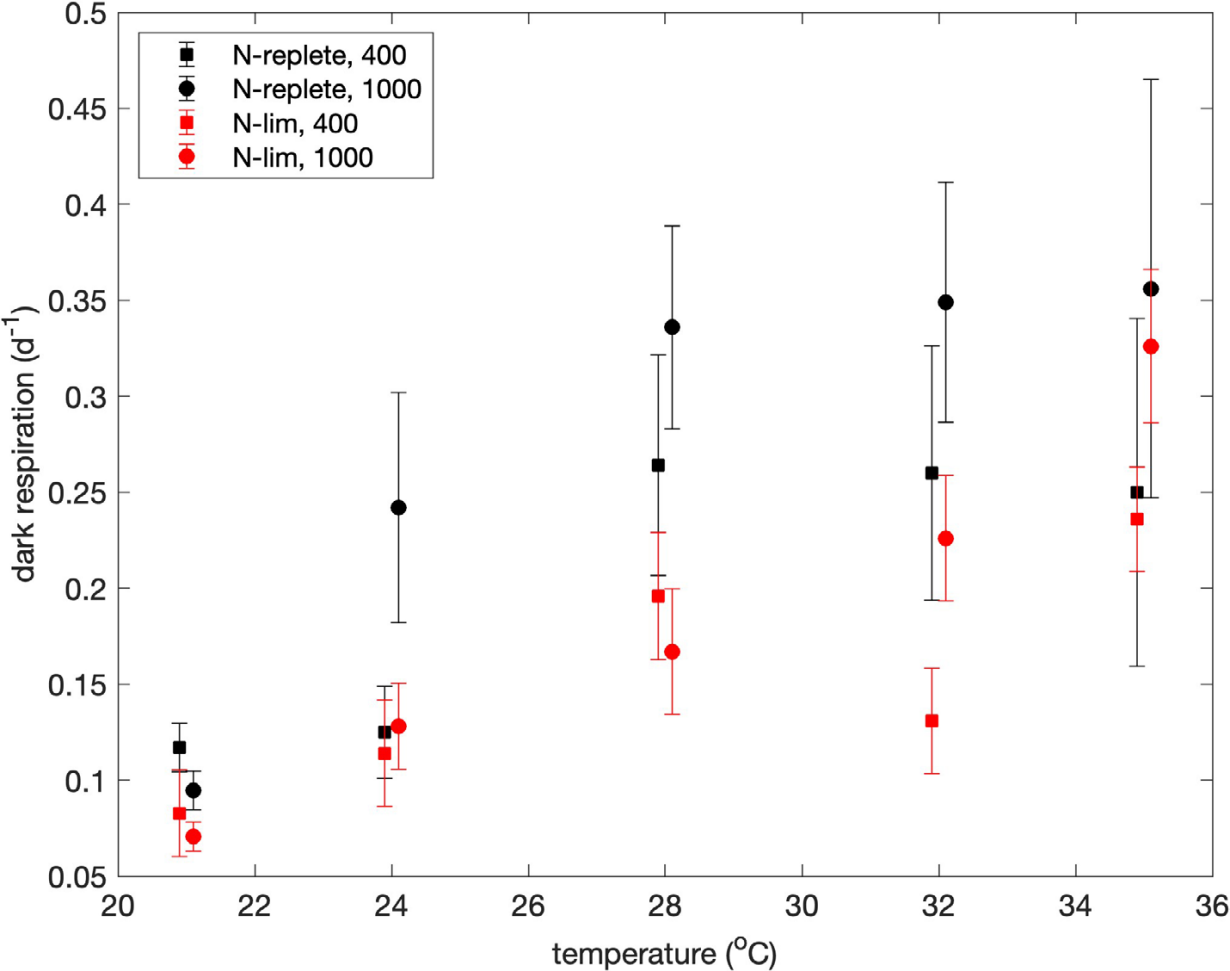
Dark respiration rates as functions of temperature, nutrient limitation, and pCO_2_. Error bars are ± median absolute deviations. Squares and circles indicate results at pCO_2_ values of 400 and 1000 ppmv, respectively. Data are offset by ±0.1°C from true temperatures to make error bars clear.

Figure 6 shows dark respiration rates as a function of temperature, pCO_2_, and degree of nutrient limitation. Paired *t*‐tests revealed significant differences between dark respiration rates at 400 and 1000 ppmv pCO_2_ (*p* = 0.019) and between nutrient‐replete and nitrate‐limited conditions (*p* = 0.0034). Median dark respiration rates were 0.164 and 0.234 day^−1^ at 400 and 1000 ppmv pCO_2_, respectively, and they were 0.255 and 0.149 day^−1^ under nutrient‐replete and nitrate‐limited conditions, respectively. The Pearson's correlation coefficient between temperature and the dark respiration rate was positive and significant under both nutrient‐replete (*r* = 0.79, *p* = 0.0062) and nitrate‐limited conditions (*r* = 0.87, *p* = 0.0012). Dark respiration rates were strongly correlated with growth rates (Pearson *r* = 0.786, *p* = 4 × 10^−5^), and the ratio of growth rate to dark respiration rate was very similar at 21°C (median = 2.86) and 35°C (median = 2.73). If respiration rates are the same during the day and night, then the ratio of net to gross production over 24 h equals μ/[2(μ + μ_*r*_)], where μ is the net growth rate and μ_*r*_ is the respiration rate. Based on the data in Table 1, this ratio was not significantly correlated with temperature (Spearman *r* = 0.12, *p* = 0.62) and averaged 0.355 ± 0.025 (median ± median absolute deviation).

## DISCUSSION

### Growth rates

There is currently much concern over how marine phytoplankton communities, photosynthetic rates, and export production in the ocean will be affected by climate change. In addition to an increase in temperature (Feng et al., 2009) and reduction in the supply of allochthonous nutrients from subsurface waters, climate change will be associated with an increase of pCO_2_, a reduction of pH (Yool et al., 2013), an increase in the concentration of bicarbonate ions, and a decrease in the concentration of carbonate ions (Iglesias‐Rodriguez et al., 2008). The impact of these changes in water chemistry on phytoplankton has been the subject of considerable discussion, experimentation, and debate (Cassar et al., 2004; Gao, Helbling, et al., 2012; Gao, Xu, et al., 2012; Goldman et al., 2017; Hopkinson et al., 2011). Uptake of both CO_2_ and bicarbonate should be facilitated by an increase of pCO_2_, but Hopkinson et al. (2011) have argued that the decrease of the energy required for carbon fixation associated with a doubling of pCO_2_ would be only 3%–6% of the energy required for carbon fixation. The NDH‐1 complex in cyanobacteria allows them to rapidly convert CO_2_ to bicarbonate and thus minimize leakage of CO_2_ from the cell (Gao et al., 2016; Price et al., 2008). Goldman et al. (2017) have argued that the reduction of seawater pH projected to the year 2100 would increase the energetic cost of maintaining intracellular pH homeostasis, and indeed, Laws and McClellan (2022) have reported that the growth rate of *Synechococcus elongatus* (CCMP 1629) decreases by a small amount rather than increases under most combinations of temperature and irradiance when the pCO_2_ is increased from 400 to 1000 ppmv.

Small genera such as *Synechococcus* with large surface‐to‐volume ratios will presumably be at a competitive advantage relative to larger genera if surface waters become more nutrient‐limited, and because the direct effect of temperature on the growth rate of strain CCMP 1334 is positive below temperatures of 35°C (Figure 2), this strain would be at a competitive advantage as the oceans warm. Because we had no success growing CCMP 1334 at temperatures above 35°C, it appears that temperatures above 35°C are supraoptimal for this strain. The results shown in Figure 2 suggest that the *Q*_10_ for the growth rate of CCMP 1334 is about 2.2 within the temperature range of 21–35°C.

Fu et al. (2007) have reported that increasing the temperature from 20 to 24°C resulted in a twofold increase in the growth rate of *Synechococcus* CCMP 1334. Their result would imply a *Q*_10_ of 5.65 for growth rate, which is more than twice the *Q*_10_ of 2.2 that we estimated from our experiments and three times the *Q*_10_ of 1.88 for phytoplankton growth estimated from both the Eppley curve (Eppley, 1972) and the quantile regression analysis of Bissinger et al. (2008). The only apparent differences in methodology between this study and that of Fu et al. (2007) were their use of semicontinuous cultures (once per day dilutions) versus our use of continuous cultures and their irradiance of 45 μmol photons · m^−2^ · s^−1^ versus our irradiance of 150 μmol photons · m^−2^ · s^−1^. The principal difference in the results is the difference in growth rates at 24°C: 0.421 day^−1^ in this study versus ~0.65 day^−1^ in the study of Fu et al. (2007). The cause of this discrepancy is unclear. We are inclined to give more credence to our results because a *Q*_10_ of 2.2 seems more plausible than a *Q*_10_ of 5.65.

The effect of roughly a doubling of current pCO_2_ levels on the growth rates of marine phytoplankton appears difficult to generalize other than the effect is almost certainly small compared with the effect of the associated increase of temperature. Our results and those of Fu et al. (2007) indicate that the growth rates of *Synechococcus* CCMP 1334 will increase if the current pCO_2_ is roughly doubled by an amount on the order of 0.1 day^−1^ that is related to temperature, but Fu et al. (2007) observed comparatively little effect of pCO_2_ on the growth rates of *Prochlococcus* CCMP 1986, whereas Laws and McClellan (2022) observed that increasing the pCO_2_ from 400 to 1000 ppmv decreased the growth rates of *Synechococcus* CCMP 1629 by small amounts except in bright light (300 μmol photons · m^−2^ · s^−1^) at the supraoptimal temperature of 45°C. The different responses of *Synechococcus* and *Prochlorococcus* to increases of pCO_2_ may be due to the absence of *Ndh* genes in the latter (Fu et al., 2007), but the different responses of *Synechococcus* CCMP 1334 and 1629 are not so easily explained.

One possible explanation for the higher nutrient‐replete growth rates of CCMP 1334 at 1000 than 400 ppmv pCO_2_ is photorespiration. Increasing the pCO_2_ from 400 to 1000 ppmv would shift the balance of competition between CO_2_ and O_2_ at the active sites of Rubisco. Laws and McClellan (2022) have likewise reported an enhancement of the growth rate of *Synechococcus* CCMP 1629 when the pCO_2_ is increased from 400 to 1000 ppmv, but in that study, the enhancement was observed only in bright light (300 μmol photons · m^−2^ · s^−1^) at the highest temperature (45°C). The enhancement we observed was greatest at the optimal temperature (32°C), when oxygen production and CO_2_ uptake by the cells would have been greatest and, hence, the effects of photorespiration most apparent. The different responses of *Synechococcus* CCMP 1334 and 1629 to a doubling of pCO_2_ may, therefore, reflect their different sensitivity to the effects of photorespiration due, perhaps, to differences between their carbon‐concentrating mechanisms.

### Carbon:nitrogen ratios

The behavior of the C:N ratio of the cells can be explained with some modification of the model of algal growth originally proposed by Shuter (1979) and later modified by Laws and Chalup (1990; see Appendix S1). According to that model, under nutrient‐replete conditions, the carbon in the cell is allocated to either structure, the light reactions of photosynthesis, or the dark reactions of photosynthesis. Under nutrient‐limited conditions, some carbon is also allocated to storage. The fraction of cellular carbon used for structure is constant. Both Shuter (1979) and Laws and Chalup (1990) assumed that the C:N ratios were the same in the structure, light reactions, and dark reactions. That assumption led directly to the relative growth rate scenario postulated by Goldman (1980). The fact that the C:N ratios were higher under nitrate‐limited conditions at the same temperature can be attributed to the carbon allocated to storage, which is assumed to contain no nitrogen. The negative correlation between the C:N ratios and temperature can be explained in the context of the Shuter (1979) and Laws and Chalup (1990) models if the C:N ratio of the biomass allocated to the light reactions is lower than the C:N ratio of the biomass allocated to the dark reactions (see Appendix S1). R‐phycoerythrin, for example, has a C:N ratio of 2, whereas the C:N ratio of microalgal protein is about 4–5 (Laws, 1991). The rationale is that there must be a balance between the rates of the light and dark reactions. The rate of light absorption is independent of temperature, but the rates of the dark reactions, which are mediated by enzymes, should be positively correlated with temperature at suboptimal temperatures. More resources must therefore be allocated to the light reactions versus the dark reactions to achieve balanced growth as the temperature is increased. Fu et al. (2007) reported no effect of temperature on the C:N ratios of nutrient‐replete cultures of *Synechococcus* CCMP 1334, but their studies were conducted at only two temperatures, 20 and 24°C. Had we based our conclusions on our results at 21 and 24°C, we would likewise have concluded that there was no effect of temperature on the C:N ratios. The effect of temperature on the C:N ratios became apparent only when the range of temperatures was extended to 35°C (Figure 3).

### Metrics normalized to Chl *a*

The pattern of C:Chl *a* ratios, like that of the C:N ratios, can be explained by the models of Shuter (1979) and Laws and Chalup (1990). The higher ratios under nitrate‐limited conditions reflects the allocation of carbon to storage products, which contain no chlorophyll, and the negative correlation between C:Chl *a* ratios and temperature reflects the need to balance the rates of the light and dark reactions of photosynthesis.

Field studies have suggested that PIs are correlated with the trophic status of marine waters (Curl & Small, 1965; Eppley, 1972; Thomas, 1970); high and low PIs are associated with eutrophic and oligotrophic conditions, respectively. Because PIs are roughly the product of the C:Chl *a* ratio and growth rate and because C:Chl *a* ratios are inversely correlated with growth rates (Figure 4), PIs tend to be insensitive to growth rates (Figure 5), as noted by (Laws & Bannister, 1980). Because C:Chl *a* ratios are negatively correlated with temperature (Figure 4), PIs also tend to be insensitive to temperature when temperatures are suboptimal.

Falkowski and Owens (1980) considered the strategies by which phytoplankton acclimate to changing irradiance. The strategies consist of changing the number of photosynthetic units or changing the size of photosynthetic units. Because phycoerythrin is the major accessory pigment in *Synechococcus*, the PE:Chl *a* ratio is a metric of the size of photosynthetic units in *Synechococcus*. Our results showed that this ratio was remarkably constant over a wide range of growth conditions (4.5 ± 0.48 g · g^−1^). The requirement to balance the rates of the light and dark reactions of photosynthesis under nitrate‐limited and nutrient‐replete conditions, 400 and 1000 ppmv pCO_2_, and temperatures of 21–35°C appears to have been satisfied in all cases by changing the number but not the size of photosynthetic units.

Fu et al. (2007) have reported that the PE:Chl *a* ratios of *Synechococcus* CCMP 1334 are higher at 24°C (6.1 g · g^−1^) than at 20°C (3.7 g · g^−1^) but did not perform a statistical test to determine the significance of the difference. Because they have reported the standard deviations of their triplicate measurements of PE and Chl *a*, we were able to generate noise‐corrupted datasets with normally distributed errors consistent with those standard deviations. Based on those noise‐corrupted datasets, we estimated the standard deviations of their PE:Chl *a* ratios to be 1.5 and 2.5 g · g^−1^ at 20 and 24°C, respectively, and therefore suspect that the difference between the ratios (6.1–3.7 = 2.4 g · g^−1^) is not significant at *p* = 0.05.

### Dark respiration rates

The increase of the dark respiration rates with temperature was very consistent with the expected effect of temperature on the rate of enzyme‐mediate reactions, and the average *Q*_10_ of 2.3 ± 0.3 was very similar to the *Q*_10_ of 2.2 for the growth rates. The failure of the cells to grow at temperatures above 35°C therefore appears to have been due to failure of gross carbon fixation at higher temperatures rather than respiration's consuming progressively more of the carbon that was fixed.

## CONCLUSIONS

Of the five hypotheses that we set out to test, we rejected the hypothesis that the nutrient‐replete growth rates of *Synechococcus* sp. CCMP 1334 would be unaffected by changing the pCO_2_ from 400 to 1000 ppmv. The growth rates were consistently higher at 1000 than 400 ppmv pCO_2_. These results were unexpected because the small size of *Synechococcus* suggests that the energy savings associated with downregulation of the carbon concentrating mechanism would be a very small fraction of the energy required for carbon fixation. The explanation may be a reduction of photorespiration due to a higher ratio of CO_2_ to O_2_ at the active sites of Rubisco.

We also rejected our second hypothesis that if pCO_2_ affects growth rates, then the effect would be independent of temperature. Although growth rates were consistently higher at 1000 than 400 ppmv pCO_2_, the difference was not statistically significant at 21°C, and it was greatest at 32°C, which was the optimum growth temperature. The dominant effect of temperature therefore obscured the CO_2_ effect near the lower thermal limit.

We accepted the null hypothesis that the C:N ratio of the cells would be independent of pCO_2_ at a fixed relative growth rate because a KW test revealed no significant effect of pCO_2_ on the C:N ratios (*p* = 0.766). However, we rejected the null hypothesis that the C:N ratio of the cells would be independent of temperature at a fixed relative growth rate. At relative growth rates of 1.0 and ~0.5, the Pearson correlation coefficient between temperatures and C:N ratios was negative and significantly different from zero under both nutrient‐replete (*p* = 0.0011) and nitrate‐limited (*p* = 0.0048) conditions (Figure 3). This behavior could be explained if the C:N ratio of the organic matter allocated to the light reactions of photosynthesis were lower than the C:N ratio of the organic matter allocated to the dark reactions of photosynthesis.

We accepted the null hypothesis that the C:N ratios, C:Chl *a* ratios, and PE:C ratios were independent of pCO_2_. KW tests or paired *t*‐tests revealed no effect of pCO_2_ in all three cases. We also accepted the null hypothesis that the variations of these ratios as functions of temperature and nutrient limitation could be explained by the model of carbon allocation by Shuter (1979) and Laws and Chalup (1990). Simplistically, the model requires that growth be balanced and that carbon be allocated between structure (constant), the light reactions of photosynthesis, the dark reactions of photosynthesis, and (in the case of nutrient‐limited growth) storage. These assumptions lead to two possible solutions, one of which maximizes the growth rate. The cells are assumed to allocate resources so as to maximize their growth rates. We accepted the null hypothesis that the productivity index (PI) would be unaffected by temperature. A KW test revealed no effect of temperature on the PI values (*p* = 0.52). We rejected the null hypothesis that the PIs would be unaffected by pCO_2_. A KW test revealed a significant difference (*p* = 0.0156) in PIs measured at pCO_2_ values of 400 ppmv (6.01 g C · g^−1^ chl *a* · h^−1^) and 1000 ppmv (7.25 g C · g^−1^ chl *a* · h^−1^). We rejected the hypothesis that the PIs would be lower under nitrate‐limited than nutrient‐replete conditions. A KW test revealed no significant difference between nutrient‐replete and nitrate‐limited PIs (*p* = 0.15).

The results of this study clearly suggest that *Synechococcus* strain CCMP 1334 would grow more rapidly in the ocean as temperatures warm and CO_2_ partial pressures increase in response to anthropogenic CO_2_ emissions. Concern has been expressed that the greater degree of nutrient limitation in a more thermally stratified water column would favor picophytoplankton relative to larger cells and hence reduce the efficiency of the biological pump. However, based on an analysis of more than 35,000 observations of the abundance of *Synechococcus*, Flombaum et al. (2013) concluded that nutrient concentrations seem to have little influence on the distribution of *Synechococcus* in the ocean. They observed that *Synechococcus* could be just as abundant in water containing 10 μM nitrate as in water containing 10 nM nitrate (Flombaum et al., 2013, figure 1b). They concluded that *Synechococcus* did not appear to be at a competitive disadvantage in water containing high nutrient concentrations. Flombaum et al. (2013) concluded that the principal determinants of the abundance of *Synechococcus* (and *Prochlorococcus*) were light and temperature. Because CCMP 1334 responded positively to warmer temperatures as high as 35°C and, to a lesser extent, to an elevation of the pCO_2_ from 400 to 1000 ppmv, this species of *Synechococcus* is likely to be favored in a warmer, high‐CO_2_ world.

## AUTHOR CONTRIBUTIONS

**Alyssa K. Sharbaugh:** Conceptualization (equal); data curation (equal); formal analysis (equal); funding acquisition (equal); investigation (equal); methodology (equal); project administration (equal); resources (equal); software (equal); supervision (equal); validation (equal); visualization (equal); writing – original draft (equal); writing – review and editing (equal). **Edward A. Laws:** Conceptualization (equal); data curation (equal); formal analysis (equal); funding acquisition (equal); investigation (equal); methodology (equal); project administration (equal); resources (equal); software (equal); supervision (equal); validation (equal); visualization (equal); writing – original draft (equal); writing – review and editing (equal).

## Supporting information


**Appendix S1.** Equations from the model of Laws and Chalup (1990) that were used to explain the C:N ratios and C:Chl *a* ratios in Figures 3 and 4, respectively.

## Data Availability

The data used to generate these results can be found in Table 1.
