# Supplementary material for: Interactive effects of CO2 , temperature, and nitrate limitation on the growth and physiology of strain CCMP 1334 of the marine cyanobacterium Synechococcus (Cyanophyceae)
Source: J Phycol. 2024 Nov 30;61(1):77–90. doi: 10.1111/jpy.13531 (PMC11914933; doi:10.1111/jpy.13531)
Supplement: Supplementary file 1 — Appendix S1. Equations from the model of Laws and Chalup (1990) that were used to explain the C:N ratios and C:Chl a ratios in Figures 3 and 4, respectively. [file JPY-61-77-s001.docx]

Appendix S1. Equations from the model of Laws and Chalup (1990) that were used to explain the C:N ratios and C:Chl *a* ratios in Figures 3 and 4, respectively.

The model described by Laws and Chalup (1990) was used to describe the variations of the C:Chl *a* ratios and C:N ratios as function of temperature and nutrient limitation. The model assumes that the carbon in the cell is allocated among four functional compartments, the light reactions of photosynthesis (P), the dark reactions of photosynthesis (E), structure S), and storage products (R). The cell is assumed to allocate carbon to these compartments so as to maximize its growth rate under a given set of environmental conditions. This requirement leads to unique specification of the carbon allocated to each of the four compartments. The fraction of carbon allocated to structure, S/C, is assumed to be constant and equal 0.1. The fractions of the carbon allocated to P, E, and R are described by the following equations:

Nutrient-replete conditions:

$\frac{P}{C}=1-\frac{S}{C}-\frac{\mu_{s}+{r_{0}}/C}{\left( 1-r_{g} \right)K_{e}}$ (A1)

$\frac{E}{C}=\frac{\mu_{s}+{r_{0}}/C}{\left( 1-r_{g} \right)K_{e}}$ (A2)

$\frac{R}{C}=0$ (A3)

Nutrient-limited conditions:

$\frac{P}{C}=1-\left( 1-F \right)\left( 1-\frac{\mu}{\mu_{s}} \right)-\frac{S}{C}-\frac{\mu+{r_{0}}/C}{\left( 1-r_{g} \right)K_{e}}$ (A4)

$\frac{E}{C}=\frac{\mu_{s}+{r_{0}}/C}{\left( 1-r_{g} \right)K_{e}}$ (A5)

$\frac{R}{C}=\left( 1-F \right)\left( 1-\frac{\mu}{\mu_{S}} \right)$ (A6)

In these equations, C is the total carbon per cell (g C ⋅ cell^−1^), μ_s_ is the nutrient-replete growth rate (d^−1^), μ is the growth rate under nutrient-limited conditions (d^−1^), r_0_/C is the basal respiration rate (d^−1^), r_g_ is the rate of change of the respiration rate per unit change of the gross photosynthetic rate (dimensionless), K_e_ is the gross photosynthetic rate per unit E (d^−1^), and F is the ratio of nutrient-limited N:C ratios at relative growth rates of 0 and 1 (dimensionless). We assumed that r_0_/C was negligible compared to μ and μ_s_, and we calculated (1 – r_g_)K_e_ as a function of temperature by comparing C:Chl *a* ratios under nitrate-limited and nutrient-replete conditions. The ratio of carbon to Chl *a* in P was assumed to be a constant, W_Chl_, and the ratios of carbon to nitrogen in P, E, and S were assumed to be constants equal to W_P_, W_E_, and W_S_, respectively, and were chosen to give a reasonable fit to the experimental data in Figures 4 and 5. The values assigned to W_Chl_, W_P_, W_E_, and W_S_ were 70, 5.6, 15.7, and 1.6 g ⋅ g^−1^, respectively. F was assigned a value of 0.25.
